# Rabies

**Published:** 1883-01

**Authors:** 


### RABIES.

[Translated from the French by the Editor.]

M. Pasteur finds in the saliva of rabies divers microbes. The inoculation of this saliva can cause death in three ways: death by the new microbe which we have made known under the name of *microbe of the saliva*, death by excessive developments of pus, death by rabies.

The *bulbe rachidien* of a person who has died of rabies, the same as of any animal that dies of rabies, is always virulent.

The poison of rabies is met with not only in the *bulbe rachidien*, but in other parts of the brain.

In order to develop rabies rapidly and surely it is necessary to inoculate the surface of the brain in the *arachnoid cavity* by the aid of the trepan.

Rabies communicated by injection of the matter of rabies in the general circulation offers frequently very different characteristics from those of the furious rage caused by biting or by trepanation, *and it is probable that many cases of silent rabies have escaped observation.*

M. Paul Bert referred to experiments he had made with the virus of rabies.

The saliva of mad dogs filtered through plaster and then inoculated remained inoffensive, while the portion remaining on top of the filter gave rabies, which proves, M. Paul Bert concludes, that this terrible malady is due to the presence of a *microbe*, as M. Pasteur has already announced. But it seems that the *microbe* of M. Pasteur has its habitual residence in the saliva, and M. Paul Bert does not recognize in the saliva the power to produce rabies. According to his view, the salivary liquid has never communicated rabies, while it does follow the inoculation of the mucous taken from the respiratory passages.

The conclusion seems to be that rabies is not produced by the saliva itself, but by the *microbes* that exist in the saliva, and that may be separated from it by filtration, leaving the saliva itself inocuous.

